# Preparation of Ultrahigh Molecular Weight Polyethylene/Graphene Nanocomposite In situ Polymerization via Spherical and Sandwich Structure Graphene/Sio_2_ Support

**DOI:** 10.1186/s11671-018-2515-4

**Published:** 2018-04-16

**Authors:** Enqi Su, Wensheng Gao, Xinjun Hu, Caicai Zhang, Bochao Zhu, Junji Jia, Anping Huang, Yongxiao Bai

**Affiliations:** 1 MOE Key Laboratory for Magnetism and Magnetic Materials, Key Laboratory of Special Function Materials and Structure Design of Ministry of Education, Institute of Material Science and Engineering, Lanzhou University, Lanzhou, 730000 People’s Republic of China; 2 Department of Material Science and Engineering, University of Florida, Gainesville, USA; 3 Lanzhou Petrochemical Research Center, Petrochina, Lanzhou, 730000 People’s Republic of China

**Keywords:** Nanocomposites, Graphene, Polymer-matrix composites, Electrical properties

## Abstract

Reduced graphene oxide/SiO_2_ (RGO/SiO_2_) serving as a novel spherical support for Ziegler-Natta (Z-N) catalyst is reported. The surface and interior of the support has a porous architecture formed by RGO/SiO_2_ sandwich structure. The sandwich structure is like a brick wall coated with a graphene layer of concreted as skeleton which could withstand external pressures and endow the structure with higher support stabilities. After loading the Z-N catalyst, the active components anchor on the surface and internal pores of the supports. When the ethylene molecules meet the active centers, the molecular chains grow from the surface and internal catalytic sites in a regular and well-organized way. And the process of the nascent molecular chains filled in the sandwich structure polymerization could ensure the graphene disperse uniformly in the polymer matrix. Compared with traditional methods, the porous spherical graphene support of this strategy has far more advantages and could maintain an intrinsic graphene performance in the nanocomposites.

## Background

Graphene, a monolayer of carbon atoms which has a tight packing of honeycomb lattice and serves as the basic building block of graphitic materials, has recently become one of the most appealing stars in material science [1]. Graphene and graphene oxide (GO), with extraordinary mechanical properties, such as high Young’s modulus, extreme hardness, excellent flexibility, and low expenses in comparison with carbon nanotube (CNT), have been considered to be effective reinforcement for high-performance composites [2–7]. Compared with conventional filled polymers, polymer/graphene composites exhibit not only the mechanical and barrier properties but also functional properties, such as electrical and thermal conductivities of polymers [8–11]. For instance, successful cooperation of graphene sheets and polymer-based nanocomposite materials has been achieved in a wide range of polar polymers, including polyaniline, polystyrene, poly(vinylidene fluoride), polyurethane, epoxy, and polythene [12–16]. However, the synthesis of graphene-reinforced polymer nanocomposites was challengeable in obtaining well-dispersed graphene sheets in polymer matrix, especially when the polymer is separated from the nonpolar polymer category typically represented by the family of polyolefin such as polyethylene (PE) or polypropylene (PP).

Generally, solution mixing is one of the ideal ways in making polymer/graphene nanocomposites if the polymer can be readily dissolved in common organic solvent. At the same time, melt mixing is also adoptable for polymer/graphene nanocomposite fabrication, which in fact is applicable to not only those solvent-resolvable polar polymers but also nonpolar polymers like polyolefin. Although the two methods have been applied to many projects, there are some drawbacks as well, for example, during the process of composite fabrication, the polymer/graphite nanocomposites could not obtain well-dispersed graphene sheets [17].

As graphene is stable and lacks functional groups, it can hardly react with the target polymer or has a good dispersion in most common solvents. It is of great interest to implement nanofiller in order to optimize the material properties. And GO platelets have been utilized as a catalyst support for the heterogeneous in situ polymerization of polyolefin, which reacts with a catalytic component by surface functional groups of GO [18]. This shows a homogeneous dispersion of few-layer GO platelets and exhibits moderate electrical conductivity [19]. In polymerization processes, the fillers are used as catalytic supports to enable the matrix to grow directly from the nanofiller surface. More nanocomposites are synthesized in this way such as clay, MMT, [20–23], and especially carbon nanotubes, which are still of great interest currently [24, 25].

Summarizing the existing reports on the preparation of polymer/graphene nanocomposites, in situ polymerization shows well-dispersed few-layer GO platelets, but composites with nonpolar polymer category like PE or PP are not able to reduce GO to graphene thermally. Catalytic components have to react with GO by surface functional groups and then obtain more severely complicated surface functional groups, which throw the graphene into disorder. The existence of defects and the disordered structure reduced some of the most important properties of graphene such as thermal conductivity, electrical conductivity, mechanical strength, and carrier mobility [26–29]. Researchers usually use the graphene layer as the catalyst to support directly for convenience, which will lead to polyolefin molecular chains growing randomly. When performing in situ polymerization, the entangling and reuniting process could easily lead to the sticky pot phenomenon. Due to the special and complicated process of the olefin polymerization, regular catalytic support, especially spherical like graphene support, is necessary. Otherwise, not only the mechanical properties of the synthesized polyolefin will degrade greatly, but also, there will be a cause of great pollution to the reactor, which will prevent the reaction works continuously and introduce unsuitable industrialization. So, the regular morphology of the graphene support is significant to the large-scale preparation of the olefin material in situ polymerization of polyolefin. To the best of our knowledge, no work for the preparation and construction of the spherical graphene-based support for the Ziegler-Natta catalyst system was reported.

To address the challenges above, we have been focusing on preparing a kind of high-efficient graphene-based support to reduce unnecessary defect generated from in situ polymerization as much as possible. The graphene-based catalyst supports act as the framework for loading catalyst in its large surface and internal pores. Meanwhile, the spherical graphene support takes the role of nanometer reinforcing filler and conductive agent. During the process of olefin polymerization, the graphene-based catalyst cracks and all the graphene layers disperse in the polyolefin matrix uniformly. Here, a unique micron spherical reduced graphene oxide(RGO)/SiO_2_ nanocomposite as support for Z-N catalyst with a novel 3D porous architecture was synthesized by the self-assembly and spray freeze-drying method. It can reach a molecular-scale homogeneity and has minimal agglomeration, and also, the size of the nanocomposite could attain 20–50 μm. In addition, we use the new support synthesis Bi-supported Ziegler-Natta catalysts of the TiCl_4_(C_4_H_9_MgCl(BuMgCl)/RGO-SiO_2_) system, and the uniform and dispersed, replicable morphology of supports, a roundish graphene/ultrahigh molecular weight polyethylene(UHMWPE) composite, has been synthesized. These properties are beneficial to continuous operation of the preparation and production equipment. Also to date, no reports have the synthesis of granuliform or roundish graphene/UHMWPE composites, which is a reappearance of the spherical morphology of the graphene-based support in the polymerization process. The nanocomposites synthesized by a Ziegler-Natta catalyst system using the micron spherical RGO-SiO_2_ supports have high-efficient graphene and reduce many unnecessary defects, which are usually generated from in situ polymerization.

## Characterizations

X-ray diffraction (XRD) patterns were recorded by a Rigaku D/Max-2400 diffractometer using Cu Kα radiation. Raman spectra of the GO, BuMgCl/GO, RGO, GO-SiO_2_, RGO-SiO_2_, BuMgCl/GO-SiO_2_, and BuMgCl/RGO-SiO_2_ were obtained with a Horiba Jobin Yvon LABRAM-HR800 with a wavelength range of 0–4000 cm^−1^. The Fourier transform infrared spectra (FT-IR) of the samples were recorded using a Nicolet NEXUS 670 FT-IR spectrophotometer, and the sample and KBr were pressed to form a tablet. The transmission electron microscopy (TEM) images were taken by the Hitachi H-600 microscope. Thermogravimetric analysis (TGA) for the samples was performed on a Perkin–Elmer diamond thermal analyzer from room temperature to 600 °C, using a heating rate of 10 °C/min with N_2_ as the sample purge gas. The fracture surface of the composites and semi-quantitative composition were analyzed by scanning electron microscopy energy dispersive X-ray detector (SEM-EDX; Hitachi S-4800). The melting point of polymers was measured by differential scanning calorimetry (DSC) at a heating rate of 5 °C/min on a Perkin-Elmer Pyris diamond thermal analyzer under the nitrogen flow rate of 30 ml/min from 25 to 400 °C. The measurement of the molecular weight was carried out on a Ubbelohde viscometer according to the Mark-Houwink equation: [η] = 1.1 × 10^−4^ M_η_^0.8^.

## Results and Discussion

### Morphology Evolution

**Fig. 1:**
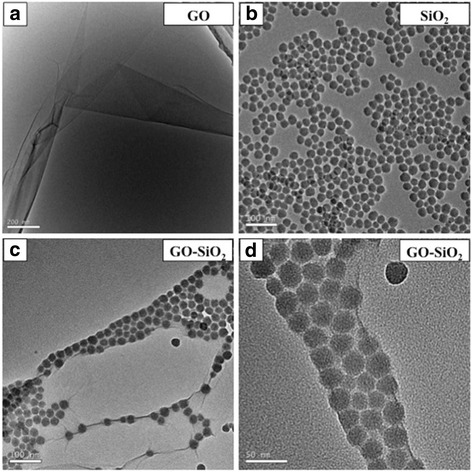
TEM images for **a** GO, **b** SiO_2_, and **c**, **d** GO-SiO_2_ hybrid

**Fig. 2:**
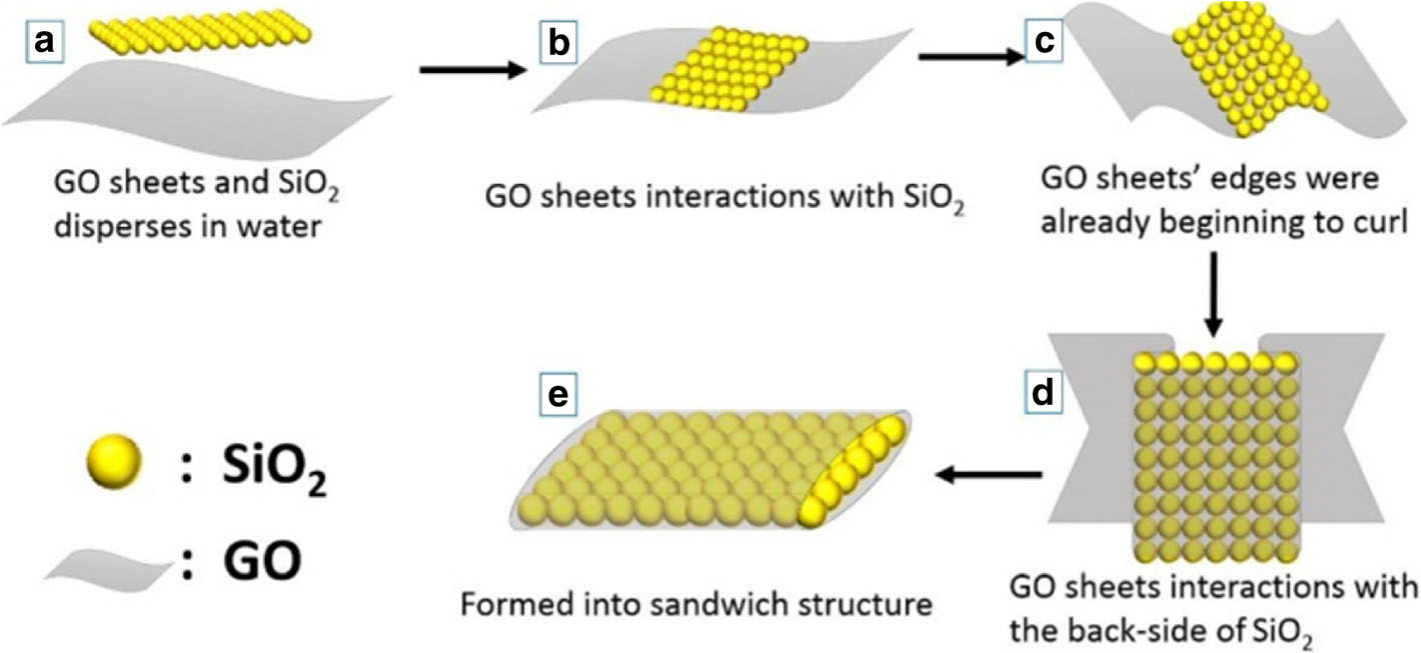
Schematic of the suggested mechanism for GO-SiO_2_ sandwich structure synthesis

Morphological changes starting from GO to GO-SiO_2_ were monitored using TEM. Figure 1a shows a TEM image of GO sheets. Figure 1b shows a TEM image of SiO_2_; the adjacent nanoparticles of SiO_2_ are easily agglomerated due to high surface energy in water. The decoration of SiO_2_ in the GO-SiO_2_ hybrid is revealed by the TEM image of Fig. 1 c, d, and it is clearly seen that the nanoparticles of SiO_2_ line up together and are wrapped in GO sheets tightly, forming a sandwich structure. The detailed pathway of building a sandwich structure from GO sheets and SiO_2_ is shown in Fig. 2.

This type of cladding layer is favorable for strong surface interactions [30]. GO is heavily loaded with oxygen-containing functional groups [31, 32] (epoxide, –COOH, and –OH); therefore, it can readily disperse in water (Fig. 2(a)) and has strong interactions with silica nanoparticles leading to the formation of GO-SiO_2_ hybrid (Fig. 2(b)) [33]. As the reaction proceeds, the edges of the GO sheets begin to curl under the great surface tension of the GO sheets (Fig. 2(c)), and then, the GO sheets interact with the back-side of SiO_2_ (Fig. 2(d)). Finally, SiO_2_ is wrapped in the GO sheets tightly, forming the sandwich structure (Fig. 2(e)); this structure helps the GO sheets avoid the risk of losing the connection and ensure electrical conductivity as a whole. On the mechanical aspect, the sandwich structure resembling a brick wall coated with a layer of concrete maintains graphene’s excellent flexibility and serves as a skeleton to withstand external pressure. Other silica nanoparticles not interacting with GO will scatter around the sandwich structure. The existence of this structure is proved by the interior structure of spherical supports and will be discussed in detail later.

**Fig. 3:**
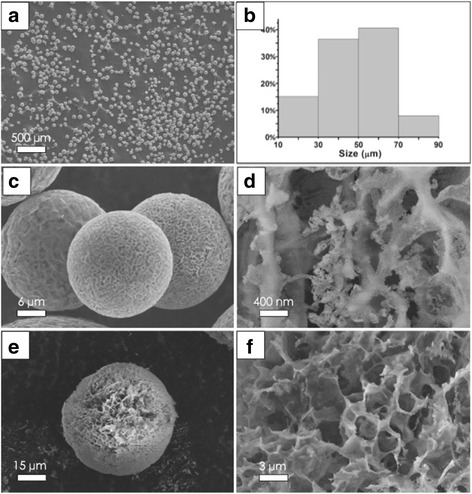
SEM images for **a**, **c** RGO-SiO_2_ spherical supports in different magnification. **b** Histograms of size distribution of **a**. **d** Surface magnification of **c**. **e** A broken sphere. **f** Magnification of **e**

SEM image in Fig. 3a exhibits the RGO-SiO_2_ supports are features of narrow size distribution, regular shape, and high porosity. And the average diameter of micro-spheres and Maken statistics of particle distribution have been calculated in Fig. 3b. The proportion of 30~70-μm-sized particles is more than 75% of all statistics, and the average particle size is 46.78 μm, the synthetized balls in accordance with the size of the catalyst supports. From Fig. 3c, it is observed that near-perfect spheres with a porous coarse cover exist, and their surface structure can be seen by the magnified image in Fig. 3d, where the porous and stacked layers of laminated structure composing of RGO-SiO_2_ sandwich structure can be found in the surface. The regular arrangement of nanospheres is seen clearly, but on the contrary, GO sheets in that the layer is too thin, the color of the sample figures the presence of GO sheets. An unambiguous broken sphere reveals that the interior is formed by a GO-SiO_2_ sandwich structure enclosing porous network structure, and the average pore size is 2.23 μm (Fig. 3f). Both surface and internal structures have great differences with the traditional silica.

**Fig. 4:**
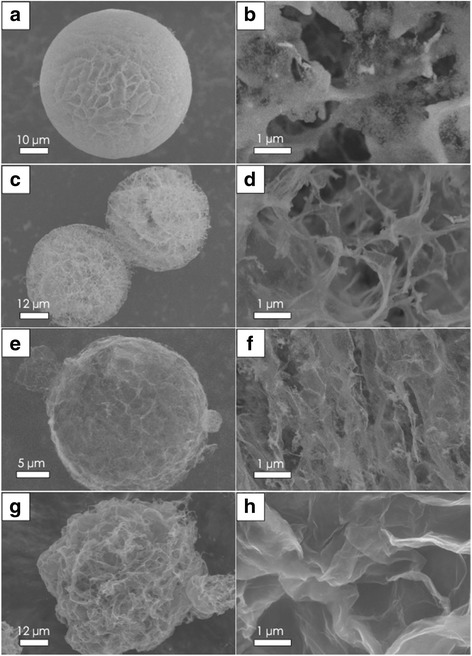
SEM images for RGO-SiO_2_ spherical supports in different SiO_2_ content. **a**, **b** Quality ratio of SiO_2_ and GO is 5:1, and magnification of **a**; **c**, **d** 1:1; and **e**, **f** 0.5:1. **g**, **h** Without SiO_2_

Studying the support morphology of the silica content, RGO-SiO_2_ spherical supports in different SiO_2_ contents are prepared by spray freeze-drying, a trend of decline sphere coefficient was observed by SEM as shown in Fig. 4.

When the quality ratio is 5:1, the morphology of the supports has not really changed too much, with some larger holes appearing on the surface and the nanoSiO_2_ losing their regular arrangement. Larger gaps have been found in the adjacent cell from Fig. 4a, b. From Fig. 4c–f, with the reduction of SiO_2_ content, some thin flaps appear at the edge of the sphere, leading to the further decline of the sphere coefficient and the scarcity of nanoSiO_2_ in many parts. From Fig. 4g, h, the material without SiO_2_ most exhibits a sphere-like shape and appears very soft much like a balloon, which demonstrates a good agreement with the experimental results. It follows the conclusion so that the addition of silica could increase the sphere coefficient and strength of the supports.

**Fig. 5:**
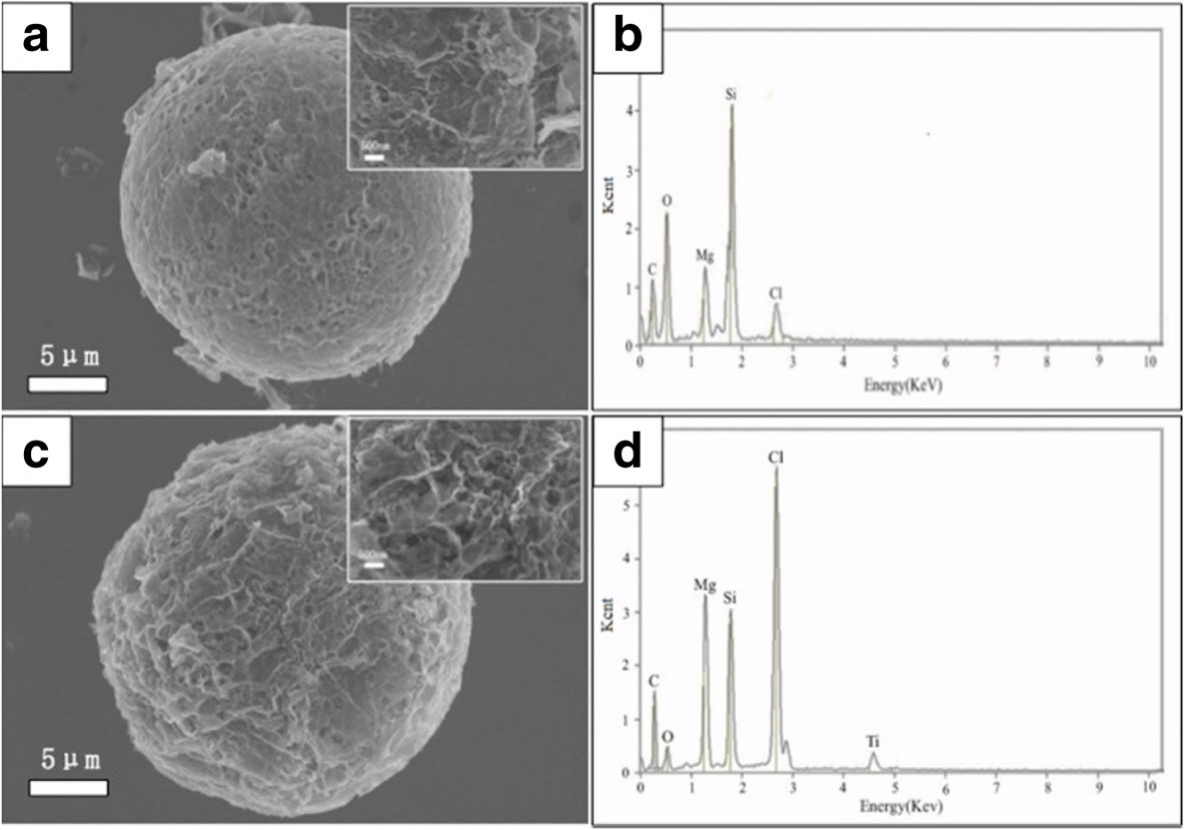
**a** SEM image of BuMgCl/RGO-SiO_2_, **b** the corresponding EDX spectrum of BuMgCl/RGO-SiO_2_, **c** SEM image of TiCl_4_/(BuMgCl/RGO-SiO_2_) catalyst, and **d** EDS spectrum of TiCl_4_/(BuMgCl/ RGO-SiO_2_) at an area indicated with a white line in part **c**

SEM image of BuMgCl/RGO-SiO_2_ is shown in Fig. 5a. Its EDX spectroscopy is presented in Fig. 5b. After treatment with BuMgCl, RGO-SiO_2_ has smaller fractured areas, because the vigorous stirring can cause collision and vibration, but the support is still a spherical porous structure, and strong peaks of Mg and Cl are presented in addition to C, O, and Si. Figure 5c shows the SEM image of the TiCl_4_/(BuMgCl/RGO-SiO_2_) catalyst, which shows that the morphology is destroyed again based on the original sphere. But the support has little impact; therefore, the overall morphology is preserved. EDX spectroscopy of the TiCl_4_/(BuMgCl/RGO-SiO_2_) catalyst in a selected area shown in Fig. 8c with a white square indicates that the transition metal Ti is supported successfully on the RGO-SiO_2_ carrier in addition to C, O, Si, Mg, and Cl (Fig. 5d).

### Defects and Disorder of Graphene in Supporters

**Fig. 6:**
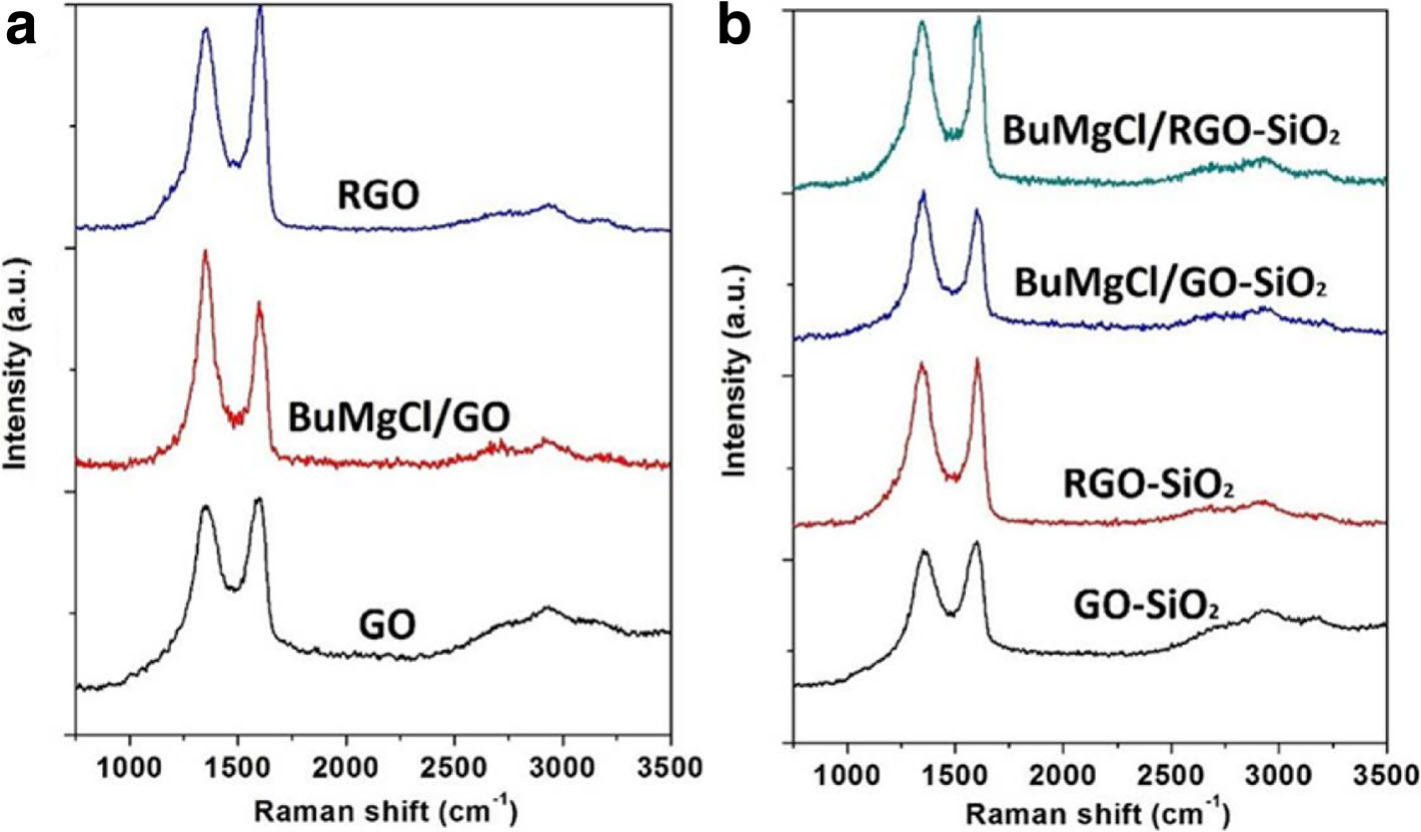
Raman spectra of GO, BuMgCl/GO, RGO, GO-SiO_2_, RGO-SiO_2_, BuMgCl/GO-SiO_2_, and BuMgCl/RGO-SiO_2_

The Raman spectra for GO, BuMgCl/GO, RGO, GO-SiO_2_, RGO-SiO_2_, BuMgCl/GO-SiO_2_, and BuMgCl/RGO-SiO_2_ are shown in Fig. 6, which consist of the G band at 1596–1607 cm^−1^ (due to aromatic structures or double bonds) and the D band at 1342–1357 cm^−1^ (associated with the order/disorder of sample). Obviously, the D band for the starting materials is relatively intense comparing to its G band, which is typical for small stacks in the defective [34]. After GO as substrate reacts with BuMgCl, Fig. 6a clearly shows that the Raman spectra is undergoing significant changes. The intensity of the D band of the resulting carbon materials intensifies sharply while BuMgCl is added, as the G band decreases, the calculated G/D ratio decreases from 1.03 to 0.78. Instead, the calculated G/D ratio of RGO increases to 1.11 while the resulting materials are heat-treated at 700 °C. This suggests a disordered arrangement of the graphene layers after GO reacts with BuMgCl. The reason is that BuMgCl reacted with the surface functional groups of GO, and these functional groups disorder the arrangement of graphene; therefore, reacting with BuMgCl have produced more severe impairment.

To solve this problem, more experiments are done in order to confirm or overturn it, as shown in Fig. 6b. When GO-SiO_2_ as substrate reacts with BuMgCl, the calculated results by this method agree well with the experiment data of GO and the G/D ratio decreases from 1.05 to 0.89. But when RGO-SiO_2_ as substrate reacts with BuMgCl, the G/D ratio decreases from 1.02 to 1.01, this is showing a clear result that an ordered arrangement of the graphene layers in the complexes is not destroyed severely after reacting with BuMgCl and SiO_2_ in the complexes are used for support BuMgCl in this process [35].

### In situ UHMWPE Polymerization with RGO-SiO_2_ Supported Ziegler-Natta Catalyst

**Fig. 7:**
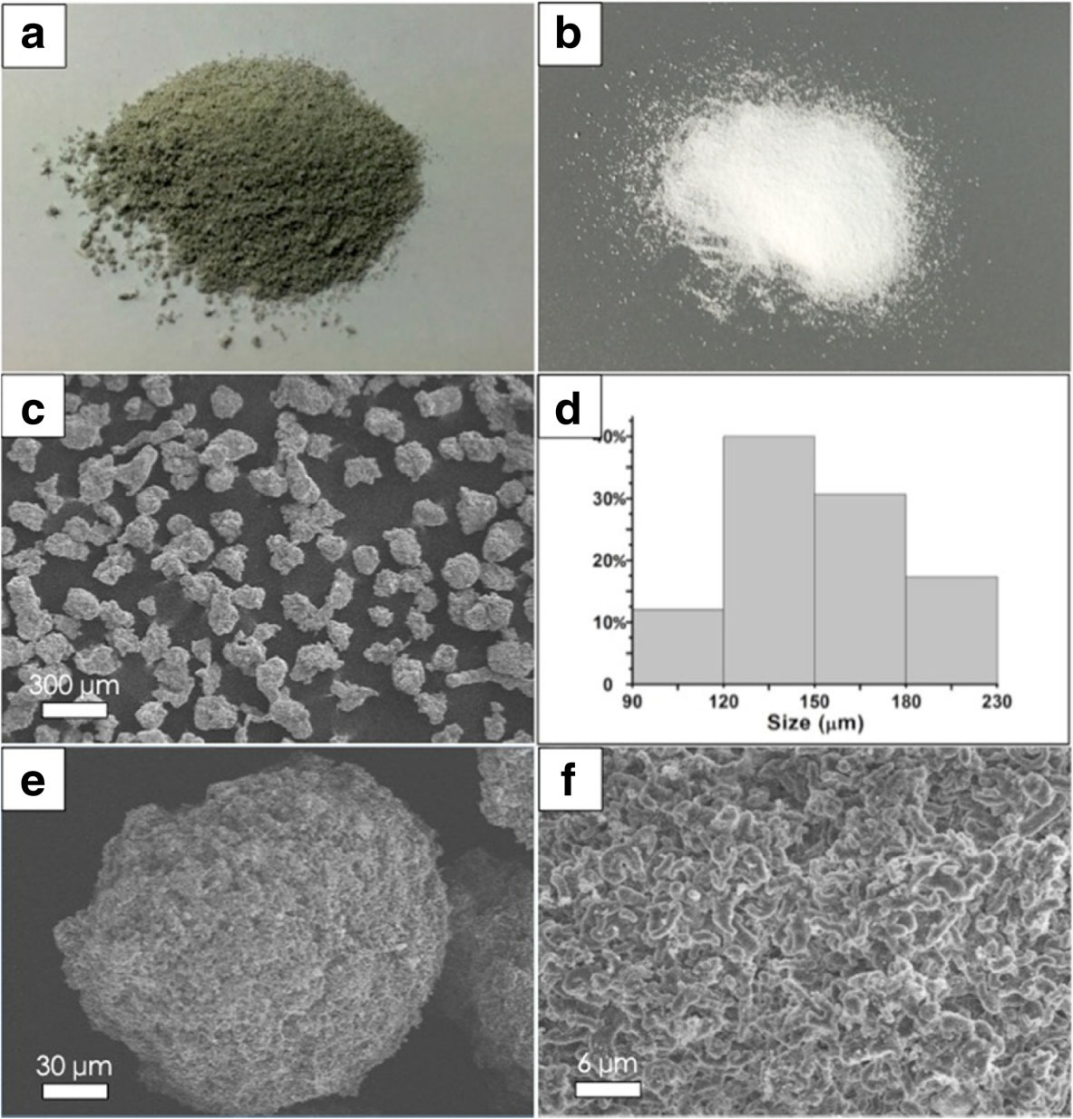
**a** UHMWPE/RGO-SiO_2_ composite powder as obtained after polymerization. **b** UHMWPE powder without RGO-SiO_2_. **d** Histograms of size distribution of **c**. **e** SEM images obtained from fracture surface of composites in different magnification. **f** Surface magnification of **e**

**Fig. 8:**
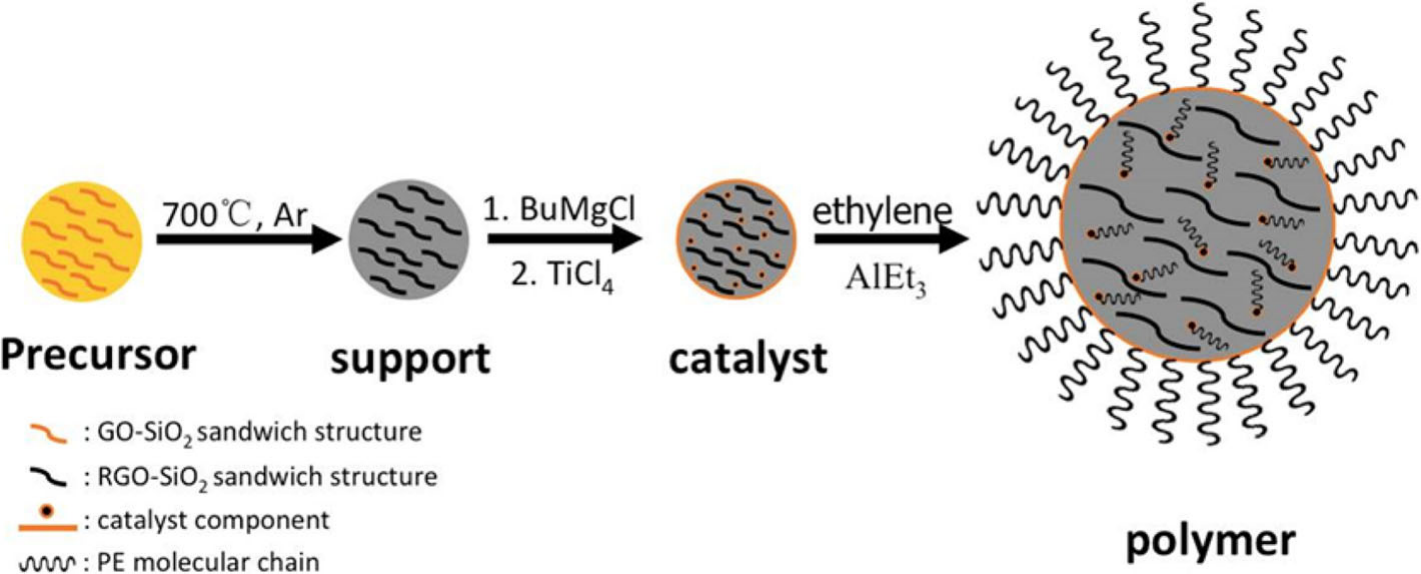
Schematic of nanocomposite synthesis

Propylene polymerization was performed with the TiCl_4_/(BuMgCl/GO-SiO_2_) catalyst. The catalyst activities were in the range of 2.66 × 10^5^–4.2 × 10^5^ g PE/mol Ti/h, depending on the triethyl aluminum [TEA]/[Ti] ratio used during the polymerization. Generally, higher [TEA]/[Ti] resulted in higher activity. DSC studied the thermal properties of the obtained UHMWPE/GO composites. A consistent polymer melting temperature near 142 °C is reassuring. The PE/GO composites with graphene loading of 0.28 and 0.5 wt% (calculated based on the added GO amount) were obtained. The samples were subjected to viscosity measurement to determine their molecular weights (M_η_). Accompanied by an increase in the amount of TEA, the viscosity-average molecular weights of the UHMWPE matrixes were changed from 3.1 × 10^6^ to 2.0 × 10^6^ g/mol, which are UHMWPE requirement. Morphological characterization of UHMWPE/RGO-SiO_2_ is shown in Fig. 7. Figure 7a shows the morphology of the UHMWPE/RGO-SiO2 composite powder as obtained after polymerization; as a contrast, the pure UHMWPE powder is white without RGO-SiO2 as shown in Fig. 7b. SEM images in Fig. 7c shows that the UHMWPE/GO-SiO_2_ composite granules have uniform size, good dispersion, and regular sphere-like shape. The average diameter of the composites has also been calculated. The statistics of particle distribution is shown in Fig. 7d and is compared with the GO-SiO_2_ supports. As the average particle size is 150 μm, which is three times of the support size, and the proportion of 120~180 μm particles is more than 70% of all statistics, that was very similar to the size distribution of GO-SiO_2_ supports, and also, the morphology of composites repeats. The detailed pathway of morphology repetition is shown in Fig. 8. From Fig. 7e, it is observed that there is a sphere particle with a cover of rough surface, and its surface structure is seen by the magnification image in Fig. 7f, the surfaces of polymer particles are almost entirely covered with graphene sheets or RGO-SiO_2_, which could explain the molecular chain growth on the surface and surroundings of catalyst.

Polymer morphology repetition based on a reaction scheme is illustrated in Fig. 8. First, the precursor, RGO-SiO_2_ spherical support, is synthesized through the reaction of thermal reduction with GO-SiO_2_ spherical composite at 700 °C in an argon atmosphere to remove residual oxygen groups internally from GO-SiO_2_ sandwich structure in the RGO-SiO_2_ sandwich structure and activate silica at the same time. Then, it reacts with BuMgCl in tetrahydrofuran (THF) to synthesize Bi support, followed by an excessive TiCl_4_ treatment to generate Mg/Ti catalyst component; a portion of component formation happens directly on the surface of support and other parts pass through the porous architecture into the internal and interact with silica nanoparticles that scattered around the sandwich structure. Then, the supported catalyst is engaged in ethylene polymerization. When ethylene met the active center, the molecular chains of the support surface grow and crystallize quickly in the surrounding of the catalyst, along with the vertical direction of spherical tangent. And the molecular chains of the internal catalytic sites also begin to grow. They filled around the RGO-SiO_2_ sandwich structure and has no interaction with graphene; therefore, graphene can disperse uniformly. With the further growing of molecular chains, the volume of polymer granules grows, and it becomes even more difficult to repeat the morphology completely. This is the reason why the nanocomposites are not near-perfect spheres in the SEM shown in Fig. 7, while the polymer has uniform size, good dispersion, and a RGO-SiO_2_ sandwich structure.

### Electrical Conductivity

The electrical conductivity of melt-compressed UHMWPE nanocomposites was measured using the four-point method on cylindrical plates with 20 mm diameter and a height of 2 mm. At 0.5 wt% GO content, the electrical conductivity was 6.46 × 10^−4^ S/cm; with decreasing GO content, it was 4.62 × 10^−5^ S/cm. Comparing to the method that used dispersed graphene nanosheet directly, this method will cost less content of graphene in forming the nanocomposites to achieve the same conductivity. The reason is that in the former method, many catalytic groups of the supported catalyst was contained on the graphene surface, then destructing the original conjugated system and deteriorating its conductivity. However, after the spherical supports are synthesized, the reaction of a Grignard reagent BuMgCl with GO-SiO_2_ in THF occurs on the support surface, which will not damage the internal graphene structure, and a conductive network is formed as the internal graphene scattered in the polymer while the polymerized samples are mixed and compressed to films.

## Conclusions

In this study, a unique micron RGO/SiO_2_ nanocomposite as a new support for Ziegler-Natta catalyst with a 3D spherical porous architecture was synthesized. We attempted to prepare the support by reducing the unnecessary defects generated during the process of in situ polymerization. The supports act as catalyst frame, reinforcing nanofillers and conductive agents. The catalytic component and active sites are uniform and well-distributed in the surface and internal pores of the supports after using the novel support synthesis Bi-supported Ziegler-Natta catalysts of TiCl_4_(BuMgCl/RGO-SiO_2_) system. And the morphologies of the polyethylene composites replicate the morphologies of the supports, then spherical UHMWPE/graphene composites have been synthesized, which is beneficial and critical to operate the continuous reparation and production equipment in the industry. In addition, these RGO-SiO_2_ nanocomposites with porous architecture also have potential applications in many other fields, such as being catalysts, energy storage materials, nanoelectronics, and photo-electronic devices.

## Abbreviations

(Z-N) catalyst: Ziegler-Natta catalyst
CNT: Carbon nanotube
PE: Polyethylene
PP: Polypropylene
RGO/SiO_2_: Reduced graphene oxide/SiO_2_
TEA: Triethyl aluminum
UHMWPE: Ultrahigh molecular weight polyethylene
